# Removing Pulps by Arsenic Pastes and Cocaine under Pressure

**Published:** 1913-12-15

**Authors:** J. W. Bryan

**Affiliations:** Nashville, Tenn.


### REMOVING PULPS BY ARSENIC PASTES AND COCAINE UNDER PRESSURE.

BY J. W. BRYAN, D. D. S., NASHVILLE, TENN.

Read before the Tennessee State Dental Association, June 6th, 1913.

In this day of advanced thinking and scientific research it has been revealed to us that micro-organisms of various kinds, qualities and conditions must be taken into account; we have germs for health and germs for sickness, and a specific germ is sought as a prime cause of each varied form of complaint. It is difficult to remain consistent and prevent some form of sepsis from creeping into what we do.

Hence the great efforts now being made for absolute cleanliness give in return assured success whenever scientifically applied, and all such methods of practice are to be commended.

It is always highly desirable that an operator shall do his best work and with the least possible risk to his patient. In order to do this he must consider well each individual case with regard to its probable pathologic surroundings, else he will get into a rut and do things from force of habit, and perhaps wonder at his failures.

A line of treatment properly applied, or applied under certain conditions may bring success, but otherwise will make conditions worse, and the operator thereby forces upon his patient the very results he has been trying to avoid. Unfavorable septic conditions may follow the removal of the pulp from a tooth as often as they are found in other lines of surgical work.

The two most generally used methods of preparing the pulp for removal are:

1st:—By the use of Arsenic to divitalize it.

2nd:—By the use of cocaine applied under pressure or otherwise to the pulp to anesthetize it.

Both of these methods are equally effective so far as the removal of the pulp is concerned, and in many cases no material difference may be seen; but when pressure anesthesia is applied to all cases the unfavorable septic results sometimes appear.

Arsenic can be applied to any pulp without any unfavorable after-effects, whether it is freshly exposed, or the exposure is of long standing, even when much of it has been removed by death or decay.

In the case of pressure anesthesia it is not safe to apply it to any pulp that is infected, or where by long exposure the pulp canal has had time for micro-organisims to gain an entrance into it, for the pressure forces the contents of the pulp canal toward and maybe through the apical foramen, and infection follows successful removal of the pulp painlessly, unless the hemorrhage from the broken tissues washes out the infection that the operator has thoughtlessly produced.

If arsenic is used in these infected pulps it destroys all forms of micro-organisms found therein, and no evil results are likely to follow, since the entire pulp canal with its contents and the dentine have been deprived of all forms of bacteria by the arsenic; but when pressure anesthesia is used, even if no infection is forced through the foramen, the pulp canal and dentine are not thus cleansed and evil results may follow unless time is taken to treat the pulp canal and thoroughly cleanse the dentine of its infectious micro-organisms.

May not this suggest a cause for teeth, whose roots have been immediately filled when pressure anesthesia has been used, giving trouble years after the work has been done? To avoid these after-evil effects it becomes our duty to study each case carefully as it is presented in order that we may select intelligently an appropiate method for removing the infected pulp. The pressure involved in any of the pressure methods is enormous, and is capable of causing infiltration of the apical tissues with the pulp contents which are of liquid consistence, or which are capable of being forced through the apical foramina. It is true also that arsenic if left too long in the pulp will dissolve and affect the apical tissues, but only in an inflammatory manner as it will not carry infection, unless the canals are left open for subsequent infection from an external source.

### DISCUSSION.

Dr. N. C. Leonard: Mr. President and Members, I do not believe that personal opinion is of very much scientific value in a discussion of a subject of this kind, and I do not believe that clinical experience is of very great scientific value. I presume every man here who has been practicing dentistry has a method that has given him pretty general satisfaction in the removal of pulps from teeth, and I doubt if anything I could say, or that Dr. Bryan has said, would very materially change his views. I think however that it is of very great importance that we arrive at some conclusion as to the real scientific method of treating these cases. Dr. Bryan seems to favor the old method of the use of the devitalizing paste. I believe it is largely a question of personal technique as to the results we get. I have no doubt that good results are being obtained by both methods. Personally, I believe in and use the infiltration of an anesthetic, and remove the pulp tissue while anesthetized. I believe the best results can be procured by this method if one is careful in the technique, no matter if the pulp is septic, or any part of the canal is septic, even though as much as a third of the root canal is still filled with living pulp tissue. A partially dead pulp affords an excellent place to apply the very strongest disinfectants we have, such as we can not use in the soft tissues, and can sterilize to a great extent the contents of the canal before the cocaine solution is applied. Then it can be applied with aseptic precautions, and by a pressure that need not force any septic material that might remain in the canal, but by a pressure that will result in the simple infiltration of the anesthetic agent in the remaining live pulp tissue. A pulp so anesthetized can be removed by a careful technique. After the pulp has been anesthetized, it seems to me we can almost preclude the possibility of infection by flooding the cavity with some strong antiseptic agent. I use oil of cinnamon, because it is rather pleasant to use, and has none of the disadvantages that others have, but any good antiseptic can be used. All instruments should be kept thoroughly sterilized, and this can be done by keeping nerve brooches in absolute alcohol until you are ready to use them. By passing the nerve instrument through a strong sterilizing agent before removing the pulp tissue, and allowing the antiseptic agent that has been used to flood the canal and forcing it into the pulp canal as soon as the pulp is withdrawn, then filling the canal with some good permanently aseptic agent we should have no infection. I prefer a paste of some kind, because that can be easily put to place. A liquid disinfectant will be displaced by gently forcing the paste of some good antiseptic agent into the canal where it will set or harden quickly. Take, for instance, the cement powder, with a little aristol, mixed with oil of cinnamon, oil of cloves or eucalyptol. This will get pretty hard, and I have never seen any evidence that it has been permeated by the fluids of the mouth or from the peridental tissues.

This is essentially the technique that I have used for perhaps fifteen or eighteen years, and I could count on the fingers of one hand all the abscesses that have come under my observation after using this method. Somebody else might have seen more of them than I have, but this is my honest observation of the results that I personally have secured. I will say that I think the difficulties of this technique and the chances of infection, seem very much exaggerated in the minds of those who have not used this method enough to become skilfull in its use.

I have not been as careful in all of the cases that I have operated on as I should, perhaps, and you will be surprised when I tell you that I very seldom use the rubber dam. I can by using cotton rolls and the saliva pump, complete the operation so quickly as to run little risk of infection. I always use strong antiseptics in the cavity, and I think I get just as good results in those cases as I have secured where I have used the rubber. Occasionally I will let my patient close his mouth during the operation. If there is a good deal of bleeding I sometimes take a syringe and wash out the cavity thoroughly. I mention this because of the suggestion I made that I think the danger of infection is somewhat exaggerated in the minds of those who have not used this method. It would be unbelievable to say that during a good many of these operations some forms of bacteria have not entered the root canal, but the treatment, and immediate root filling with a strong antiseptic and disinfectant paste seem to take care of all infectious material in practically all cases and also of any bacterial element that may have entered the canal during treatment.

From my experience I do not believe it is hardly possible in an operation of this kind, to force bacteria through the pulp tissue and out the tissues into the peridental tissues.

These are just my personal opinions and are not of any scientific value,—I realize that. I wish somebody would devise some method of proving these things absolutely as it seems it could be done. On the other hand, I believe that all of us have observed cases where considerable damage has resulted from the use of arsenic, even by a small application of arsenic. I think the more scientific method would be one that is in line with all modern surgical methods. The surgeon does not devitalize the member and then remove it, but he does it with a plain, well defined surgical operation that separates the healthy living tissues, and then places dressings that are not irritating to the same, but will protect it from the invasion of any bacterial element.

Dr. John Beach: I believe that the performance of this operation with the same care that a surgeon would perform a similar operation, we should expect to have perfect success in the removal of any pulp. If there is hemorrhage we can arrest that, and fill the root canal immediately, and I believe we should have better results than if we operate or devitalize and rely on any methods of sterilization afterwards, especially if we insist on having properly sterilized instruments and an aseptic procedure.

Dr. Ewell Neil: We all know there are many cases where we desire to destroy the pulp that don't call for a choice of methods. For instance, in case of hyper-sensitive dentine, you cannot grind down the tooth in preparing it for an abutment without devitalizing it, and frequently these teeth are so sensitive that the patient will not even allow us to make a cavity in which to place the devitalizing agent. I have placed cotton carrying the devitalizing agent on the surface of the tooth, and put over that very thin cement, and by infiltration the arsenic will pass through to the pulp and destroy the vitality of pulp in that way, is there not an element of danger in those cases where we have an exposed pulp, and where we place the devitalizing agent in the pulp chamber, that the arsenic will pass through the pulp to the peridental tissues? This has occurred to me many times, but I have never heard it discussed before.

Dr. L. G. Noel: Right now I want to make an argument against using only one agent. I have argued the matter out with my friend Leonard a good many times, and we do not get any nearer to a conclusion. I should not like to be confined to cocaine. In the case just cited, and in a good many cases where you can not get near enough to an exposure to anesthetize the pulp you want to use arsenic to get into that dentine and get it exposed. It has its use right there. Dr. Spooner introduced arsenic as an agent for devitalizing pulps years ago, and it has been a good friend to the profession ever since, and I would not like to be deprived of it. I used it the other day on my friend Neil. He thought he had some toxic effects from the cocaine. I want to ask the question if those of you who are using cocaine often meet with that trouble? Carelessly we go along and use this pressure anesthesia without the aid of the rubber dam, or without suggesting to the patient, that he be careful not to swallow any of the drug, and he forgets himself and swallows some with unfavorable results. Is it possible for us to get the toxic effect of the drug through the tissues by pressure? I have had several cases where my patients have felt the peculiar benumbing effects in the extremities. I always caution my students to put on the rubber dam in these cases, and to be careful to caution the patient not to swallow. I think we ought always to sterilize the mouth of the patient as much as possible, and select the patients for cocaine and arsenic with judgment, and not deny ourselves the privilege of selection, by saying, I will not use this or that. Use anything you can that will give the best result.

Another argument against my friend Leonard, and I love to have arguments with him because he likes it himself, is in a great many cases we have molar teeth with the pulp dead in one root and living in another, and where it is dead, organisms have already found their way well up in the apical regions. I believe it possible for us, in using cocaine pressure anesthesia in a case of that kind to force septic matter through the foramen. How are you going to prevent it? Clean out the canal with a dead pulp and fill it, then use the cocaine with pressure in the canal containing the living pulp. I doubt whether disinfecting agents like creosote, or bichloride of mercury if placed for several days in such a tooth, would effect the apical space.

Dr. H. A. Holder: In answer to Dr. Noel's question, Dr. Noyes, in his recent work says he has experimented to see just how much connection there was between the dental tubuli and the pericementum, by sealing medicaments into roots of teeth, and placing those teeth in water for a number of days, and then having a chemical analysis made of the water. In no instance has he been able to demonstrate the presence in the water of the medicaments placed in the canals. If true, I think that would dispose of the question in regard to the penetration of arsenic. I think the only case where the pericementum would be reached, would be where an excessive amount of arsenic was used, or where sealed with a temporary stopping in a case in which the patient exerted a pumping force in the mastication of food thus forcing it through the foramenina.

Dr. D. M. Cattell: I want Dr. Bryan to explain positively whether he meant to say that in placing an arsenical treatment in a root canal that is infected, that the arsenic kills not only the pulp, but the germs that are within. I did not know before that arsenic was a germicide in that sense. I have never seen or heard that it was placed in the list of germicides, and if it is true that arsenic is a germicide, taking hold of the bacteria that are already in the canal or through the pulp, and will kill them as readily as the pulp, then it has a new mission I did not know of.

Dr. Eugene Johnson: I use all of these methods. I use arsenic and some cocaine, very little cocaine, because I have noticed some of the trouble Dr. Noel mentions with cocaine. I have used novocaine which is ten times less toxic than cocaine, and I used it by infiltration, not through the pulp canal, but through the alveolar process. For the lower teeth, especially the posterior teeth, I go back to the entrance of the inferior dental nerve into the canal, about on a line with the morsal surfaces of the teeth, to infiltrate the nerve trunk. This anesthetizes all the lower teeth, and I have never had any bad results from this treatment. For the upper I drill into the alveolar process back of the tooth with a No. $\frac{1}{2}$ round burr, and inject the anesthetic, and you can excavate to the pulp all right, without pain, and have no trouble at all. I have been using this for three and a half years, and very seldom have to resort to cocaine anesthesia. I do not use it when the gums are in such bad condition that I am afraid to make an incision.

Dr. C. H. Taylor: I want a minute to answer a question that I understood Dr. Noel to ask—whether or not any of us have found we got constitutional effects from the use of cocaine, or pressure anesthesia, through the nerve canals. I beg to state that I have had numerous cases where my patients complained of tingling sensations running down to the ends of the toe . One case especially, of a child eight or nine years old, with an exposed pulp in the upper first molar, I finally used pressure anesthesia and removed the pulp. By the time I had gotten through removing the pulp I was to extract the second temporary molar next to it—the patient had worried me so I didn't care if I did hurt him a little bit, so I slipped up and extracted that molar without letting him know what I was doing, and I discovered that I had driven the cocaine through to the apex sufficiently to anesthetize and remove the tooth without pain.

Dr. J. W. Bryan: My paper has had a better discussion than I anticipated. My only aim was to excite interest in this subject so that each one would be so impressed with its importance, that he will go back home and study his individual cases more carefully and see what is best to do. I have no other axe to grind.

I agree with Dr. Leonard that in many cases—not always, but in many cases—I use pressure anesthesia with absolute success and satisfaction, so far as I know, but in those cases I must be fully convinced of the fact that the pulp canal does not contain septric micro-organisms, either in the pulp or in the fluids that are contained within the pulp canals. If you are to remove a pulp from a sound tooth, and can expose the pulp, that is an ideal case for pressure anesthesia, because no micro-organisms, no sepsis of any kind, is in that pulp canal, nothing detrimental except what you may introduce through a lack of proper care.

But the question I want to bring out is this—when you dig out a pulp canal, say the pulp is half dead—not only the pulp, but the fluids that are in that pulp canal are just burdened with these micro-organisms and ptomains that produce all forms of infection. How on earth I can put pressure in that canal and not force these fluids through the apex, I can not see. Why, it is about like using your hyperdermic syringe, loaded with fluid, and making pressure on the piston, and none of it passing through the point of the needle. This is one thing I wanted to bring out. If I could do it safely, that is the way for me to do it. If you can, that is the way for you to do it,—but you must keep everything thoroughly aseptic. I can agree with almost everything that has been said, but we were taught, I believee by Dr. Noel, when I was here in school, that these little micro-organisms would pass into the dentine, and unless some disinfecting was done, diseased dentine was the result. Unless we get the root of the tooth filled with an antiseptic treatment, so that further infection can not be set up, some time later these teeth, under peculiar conditions, become infected and alveolar abcess results. May not this be attributable to the fact that we left some of those little micro-organisms in the dentine? May not that account for the trouble resulted after the lapse of years perhaps?

I don't believe it is possible, unless you completely saturate the entire tooth which is so completely infiltrated with bacteria to render it thoroughly sterile by any applications to the pulp canal.

Dr. Cattell asked if arsenic is a germicidal agent. It is a known fact that tissues that have been thoroughly saturated with arsenic have been preserved for many years without further decomposition. I have taken pulps and put them in a solution of arsenic and laid them aside and kept them indefinitely, without any tendency toward further decomposition. Whether it is germicidal beyond this I do not know.

